# Zonation of Landslide Susceptibility in Ruijin, Jiangxi, China

**DOI:** 10.3390/ijerph18115906

**Published:** 2021-05-31

**Authors:** Xiaoting Zhou, Weicheng Wu, Ziyu Lin, Guiliang Zhang, Renxiang Chen, Yong Song, Zhiling Wang, Tao Lang, Yaozu Qin, Penghui Ou, Wenchao Huangfu, Yang Zhang, Lifeng Xie, Xiaolan Huang, Xiao Fu, Jie Li, Jingheng Jiang, Ming Zhang, Yixuan Liu, Shanling Peng, Chongjian Shao, Yonghui Bai, Xiaofeng Zhang, Xiangtong Liu, Wenheng Liu

**Affiliations:** 1 Key Laboratory of Digital Lands and Resources and Faculty of Earth Sciences, East China University of Technology, Nanchang 330013, China; 201900818004@ecut.edu.cn (X.Z.); zylin@ecut.edu.cn (Z.L.); qyz60010@ecut.edu.cn (Y.Q.); 201810818013@ecut.edu.cn (P.O.); 201810818004@ecut.edu.cn (W.H.); 201810818002@ecut.edu.cn (Y.Z.); 201810705007@ecut.edu.cn (L.X.); 201810705009@ecut.edu.cn (X.H.); 201910705015@ecut.edu.cn (X.F.); 201910705016@ecut.edu.cn (J.L.); 201910818023@ecut.edu.cn (J.J.); 201910818003@ecut.edu.cn (M.Z.); lyx201910853001@163.com (Y.L.); pshanling@ecut.edu.cn (S.P.); scj350936@ecut.edu.cn (C.S.); 201960070@ecut.edu.cn (Y.B.); 6owenlwh@163.com (W.L.); 2 264 Geological Team of Jiangxi Nuclear Industry, Ganzhou 341000, China; zgl-63@163.com (G.Z.); crxkcy@163.com (R.C.); songyong1109@126.com (Y.S.); deva84@163.com (Z.W.); ecitlangtao@163.com (T.L.); 3 School of Geophysics and Measurement-Control Technology, East China University of Technology, Nanchang 330013, China; 201160023@ecut.edu.cn; 4 Faculty of Geomatics, East China University of Technology, Nanchang 330013, China; 200760007@ecut.edu.cn

**Keywords:** landslide, geo-environmental factor quantification, random forest, susceptibility zoning

## Abstract

Landslides are one of the major geohazards threatening human society. The objective of this study was to conduct a landslide hazard susceptibility assessment for Ruijin, Jiangxi, China, and to provide technical support to the local government for implementing disaster reduction and prevention measures. Machine learning approaches, e.g., random forests (RFs) and support vector machines (SVMs) were employed and multiple geo-environmental factors such as land cover, NDVI, landform, rainfall, lithology, and proximity to faults, roads, and rivers, etc., were utilized to achieve our purposes. For categorical factors, three processing approaches were proposed: simple numerical labeling (SNL), weight assignment (WA)-based and frequency ratio (FR)-based. Then 19 geo-environmental factors were respectively converted into raster to constitute three 19-band datasets, i.e., DS1, DS2, and DS3 from three different processes. Then, 155 observed landslides that occurred in the past decades were vectorized, among which 70% were randomly selected to compose a training set (TS1) and the remaining 30% to form a validation set (VS1). A number of non-landslide (no-risk) samples distributed in the whole study area were identified in low slope (<1–3°) zones such as urban areas and croplands, and also added to the TS1 and VS1 in the same ratio. For comparison, we used the FR approach to identify the no-risk samples in both flat and non-flat areas, and merged them into the field-observed landslides to constitute another pair of training and validation sets (TS2 and VS2) using the same ratio of 7:3. The RF algorithm was applied to model the probability of the landslide occurrence using DS1, DS2, and DS3 as predictive variables and TS1 and TS2 for training to obtain the SNL-based, WA-based, and FR-based RF models, respectively. Verified against VS1 and VS2, the three models have similar overall accuracy (OA) and Kappa coefficient (KC), which are 89.61%, 91.47%, and 94.54%, and 0.7926, 0.8299, and 0.8908, respectively. All of them are much better than the three models obtained by SVM algorithm with OA of 81.79%, 82.86%, and 83%, and KC of 0.6337, 0.655, and 0.660. New case verification with the recent 26 landslide events of 2017–2020 revealed that the landslide susceptibility map from WA-based RF modeling was able to properly identify the high and very high susceptibility zones where 23 new landslides had occurred, and performed better than the SNL-based and FR-based RF modeling, though the latter has a slightly higher OA and KC. Hence, we concluded that all three RF models achieve reasonable risk prediction, but WA-based and FR-based RF modeling deserves a recommendation for application elsewhere. The results of this study may serve as reference for the local authorities in prevention and early warning of landslide hazards.

## 1. Introduction

Landslides are frequent natural disasters, which pose a serious threat to transportation, property, and safety of people [1,2,3,4]. Petley (2012) stated that the damage caused by non-seismic landslides is severe around the world, especially in Asia, and we previously underestimated the toll caused by this type of disaster [5]. Ruijin County in Jiangxi, South China, is such an area where landslide calamity constitutes a serious threat and problem to human society. According to the 264 Geological Team (of the Geological Bureau of Jiangxi Nuclear Industry), landslides have caused damage to 104 residential buildings, and made 2319 people homeless in the past decades. Affected by a landslide disaster, the construction and use of the newly-built campus of No. 6 Middle School of Ruijin was suspended. The uncertainty and suddenness of landslide disasters constitute potential threats to human daily life [2,4]. Therefore, recognition of potential landslide-prone areas is an essential part of hazard early warning systems, which aids decision-makers in land use planning and resource management, and reduces losses caused by disasters [6,7,8].

In the past decades, a number of studies about landslide susceptibility prediction and assessment have been conducted [6,9,10,11,12,13]. These studies proposed a variety of landslide susceptibility prediction and assessment methods, e.g., knowledge-based, physical, and data-driven methods [14,15,16]. However, advantages and limitations exist in each approach, for example, knowledge-based and physical methods are mostly intuitive but qualitative or half quantitative [14,16,17], while data-driven methods are quantitative, yet require powerful computing capacity for big data processing. Overall, data-driven methods seem more promising for a higher prediction accuracy than other methods, and thus, are more suitable for landslide susceptibility assessment in areas where there is insufficient geotechnical data [10,14,16,18,19,20].

Due to the heterogeneity in geological and environmental conditions, the scale and mechanism of landslides may differ from one place to another [21]. This makes hazard prediction complicated and requires consideration of as many of the hazard-causative factors as possible while dealing with susceptibility assessment. Recently, remote sensing (RS) and geographic information system (GIS) have been taking an active part in the study of disaster risk zoning [22,23,24,25,26]. RS techniques can not only provide multitemporal and time-series spatial information of large and even inaccessible areas over a span of decades but also timely pre- or post-hazard spatial data [8,27,28]. Therefore, RS is an effective tool for hazard monitoring and assessment. To be precise, satellite images can provide important environmental factor layers (e.g., topography, land cover, and anthropogenic activities) which can be used for landslide susceptibility prediction and modeling [8,29]. The other main geological, soil, and meteorological hazard-prone factors are also fundamental and essential for this purpose [8,17,21].

In the past years, artificial intelligence, notably, machine learning techniques including deep learning have gained a momentum in geospatial big data processing. For example, data-driven algorithms such as support vector machines (SVMs), random forests (RFs), and artificial neural networks (ANNs) have been well applied in land cover mapping [7] and prediction of soil salinity [30] and ore mineralization [31] in geological fields, and shown superior performance to traditional approaches [32,33,34,35,36]. Comparing with other machine learning approaches, the RF algorithm has clear advantages, i.e., it does not require the data to be normalized and discretized, is less sensitive to outliers, and runs faster than SVMs [7,37,38]. Landslide causative factors often present nonlinear relationships [14,19]. The RF algorithm can catch such nonlinear features among the factors but prevents overfitting [8,17,37]. Hence, the reliability of landslide susceptibility prediction mainly depends on the modeling approaches and the selection and processing of the available hazard-causative factors [39,40,41].

In view of the reliable prediction result obtained from regression and classification with the RF algorithm [7,30], the objective of this study is to employ this algorithm, one of the data-driven methods, to model the landslide susceptibility taking Ruijin, Jiangxi as an example. One specific objective of this research is to find out which kind of processing approaches for quantification of the categorical variables and for selection of the no-risk (stable) samples will best help predict the landslide susceptibility, and which algorithm, between RF and SVM, is more suitable for achieving a reliable prediction.

## 2. Data and Methods

### 2.1. The Study Area

**Figure 1:**
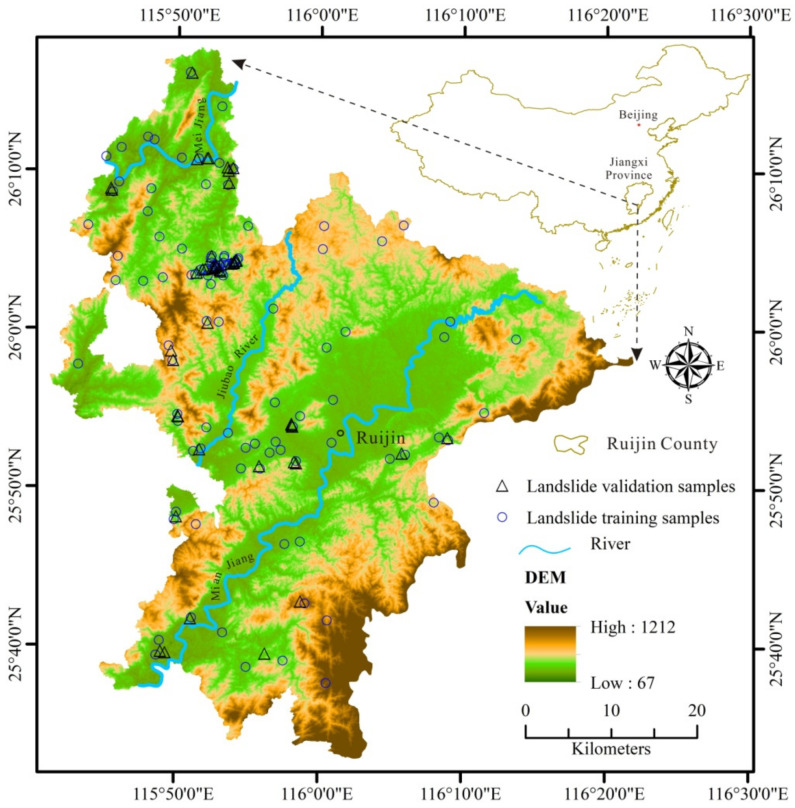
Location of the study area, Ruijin County, Jiangxi, China, and location of the training and validation sites of landslides in the study area.

Ruijin is located in the southeast of Jiangxi Province, China, extending from 115°41′10″ to 116°21′49″ E in longitude and from 25°32′15″ to 26°17′45″ N in latitude, covering an area of about 2436 km^2^ (Figure 1). Topographically, the elevation of the study area varies from 70 to 1211 m with a mean of 324 m while the slope varies from 0 to 65° with an average of 15°. Hydrologically, the main rivers are Meijiang, Mianjiang, and Jiubao, which run through the study area as sub-tributaries of the Gongshui River watershed. The study area belongs to the subtropical humid climate zone and is characterized by four distinct seasons, sufficient rainfall, and a long frost-free period. Heavy rainfall often occurs from April to July, accounting for 65.45% of the annual rainfall with amounts of about 1663.5 mm, an average of the period from 1968–2017. The annual mean temperature is 21.54 °C and July is the hottest month of the year with a mean temperature of 28.8 °C.

The hot and humid weather leads to severe weathering of rock mass giving rise to formation of a thick weathered crust in which most landslides take places. On the human side, artificial cutting of slopes for infrastructure construction (such as roads and highways) and housing development provokes instability of the crust mass, causing landslides.

### 2.2. Field Survey Data

During the first field investigation, 155 historical landslides that had occurred in the period of 2000–2017 were collected by the 264 Geological Team from 2014–2017. Our field investigation conducted from July 2019–August 2020 recorded 26 new landslides including one rockfall and two large-scale bedding slides.

#### 2.2.1. Observed Mechanisms 

It came to our knowledge that there are three main types of landslide mechanisms in Ruijin: (1) the majority of the slides occur in the weathered crust along certain unconformity surfaces or in the talus without clear sliding surface, mostly small in scale caused by road construction; (2) very local rockfall; and (3) large-scale downhill bedding slide, or creeping block slide that causes geological formations together with the overlying regolith mass to slowly slide downward. The rotational landslides and debris flow were not found [42]. The creeping downhill bedding slides take place in the Carboniferous and Cretaceous strata in which sandstone and shale or mudstone are interbedded with each other and the bedding serves as a slide surface. The measurement illustrated that the bedding dip is mostly <10–20°, which is where the creeping landslides that threatened and damaged the newly built No. 6 Middle School of Ruijin and the Longzhu Temple developed (see Discussion).

#### 2.2.2. Triggering Factors

Among the aforementioned geo-environmental factors, geological strata and their lithologies, faults, development of joints, slope degree, and so on are the inherent factors of influencing the landslide gestation. Whereas, strong rainfall and river cutting are the exogenous environmental triggering factors, while human activity, e.g., road system and housing development, is the artificial triggering factor that modifies the landscape and destroys its original balance. Notable, about 94.8% of the observed landslides are distributed along the two sides of roads and on the fringe of newly urbanized areas. Hence, most of the landslides are a result of the combined action of road and house construction and rainfall.

### 2.3. Data and Processing Procedures

For the landslide susceptibility assessment, it is unavoidable to deal with both numeric and categorical geo-environmental factors such as rainfall, slope, geological strata, faults, and rivers as they are essential for this purpose. Hence, it is necessary to convert the categorical factors into numeric or meaningful values so that they can be incorporated as quantitative variables for landslide susceptibility modeling by RF and SVM algorithms. We introduced two approaches for this conversion, i.e., simple numeric labeling (SNL) and weight assignment (WA).

**Figure 2:**
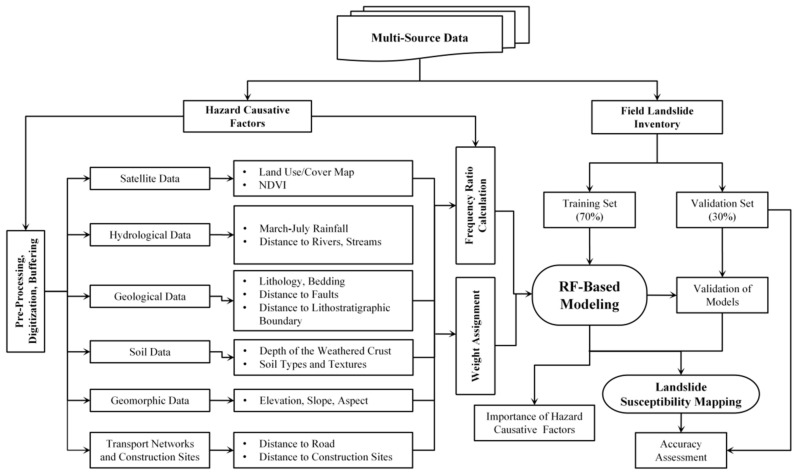
Methodological flowchart.

The global methodological procedure includes data pre-processing, digitization, linear features buffering, rational numeric value assignment to descriptive factors and buffers, susceptibility modeling and validation, and finally, accuracy assessment. These procedures are presented in a flowchart shown in Figure 2.

#### 2.3.1. Geo-Environmental Data and WA-Based Processing

##### Satellite Data

**Figure 3:**
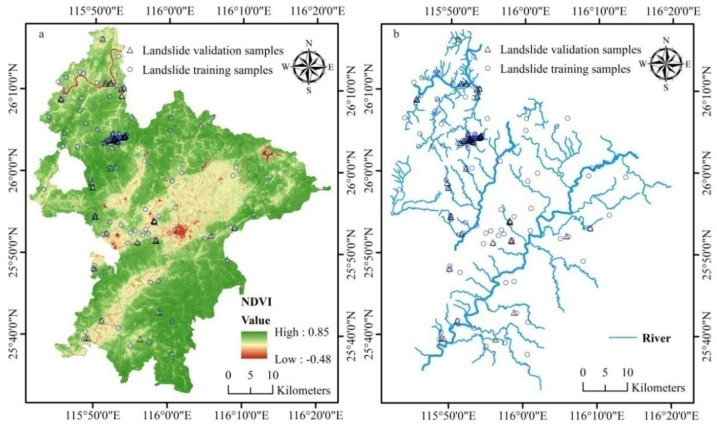
Geo-environmental factors 1: (**a**) NDVI and (**b**) rivers.

(1) Landsat imagery: Landsat 5 TM images of late October and early November from 2006–2010 and Landsat 8 OLI images dated May 2017 and Sept 2019 were obtained from the USGS data server (https://glovis.usgs.gov, accessed on 20 May 2020). After atmospheric correction using the COST model [23,43,44], Landsat 8 images were employed for land cover mapping using the approach proposed by Wu et al. (2016) [7] and Landsat 5 data for deriving the averaged multiyear autumn NDVI (Figure 3a).

Here, NDVI represents the coverage and vigor of forests and woodlands as crops have been harvested and herbaceous vegetation has become withered in late autumn. In general, vegetation, especially trees, can help soil hold water content and improve its mechanical properties through root systems which stabilize slopes. Thus, landslides may arise more likely in unvegetated areas rather than in forests and woodlands [45,46]. Slope cutting and excavation for road construction and housing exacerbate the susceptibility even in areas with vegetation.

**Figure 4:**
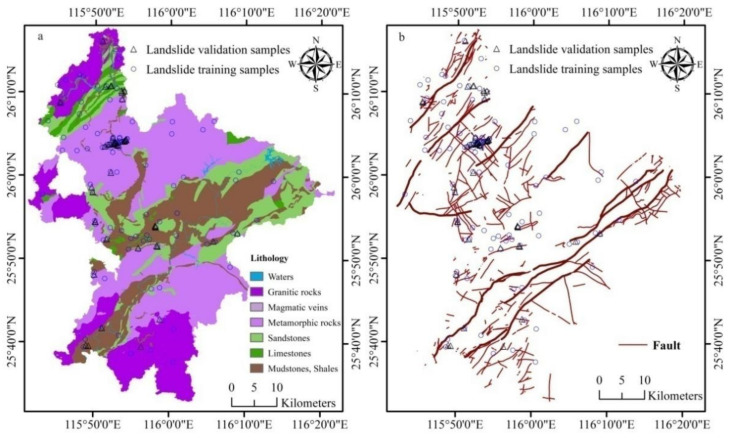
Geo-environmental factors 2: (**a**) lithology and (**b**) faults.

(2) Very high-resolution images, available on Google Earth (©Google), were used as a complementary source of ground-truth data. The road and river networks were also extracted from Google Earth (Figure 3b and Figure 4d).

According to the principle of the machine learning algorithm, we used two types of samples for modeling as input variables: one is the locality of landslides that have taken place and the other is the stable areas where landslides are unlikely to occur [8,47,48]. Identified on Google Earth, the stable areas are places where the slope is less than three degrees, e.g., water bodies, urban areas, and cultivated land. Landslides with an area greater than 900 m^2^ (1 Landsat pixel) that were overlooked during the field observation were also identified and delineated on Google Earth.

##### Hydrological Data

(1) Rainfall: Monsieurs et al. (2018) and Depicker et al. (2020) stated that rainfall was the direct cause, or rather, the triggering factor of many landslides [38,48]. Daily rainfall data from January 2008 to December 2013 were obtained from 40 meteorological stations in Ruijin and its adjacent areas. As the landslides mainly occurred in March to July, especially, in June and July but without detailed recorded occurrence time, our intention was to investigate which months of rainfall or their combinations may best reveal its role in landslide events. Thus, apart from the mean annual rainfall, March-June, May-July and March-July rainfalls of these six years were also aggregated and gridded into raster with 30 m pixel size using the inverse distance weighting (IDW) approach.
(2) River network: The influence of rivers on the occurrence of landslides is reflected by the proximity to, or rather, distance from rivers [21,49,50]. Thus, the rivers were vectorized from Google Earth (Figure 3b) and buffered into belts with an interval of 30, 60, 90, 120, and 150 m, respectively, for streams, and 60, 120, 180, 240, and 300 m, respectively, for the main rivers. Then, these buffers were assigned values in terms of their propensity or their importance in the event of a landslide based on the field knowledge and expert judgment. For example, for the main river buffers of 0–60, 60–120, 120–180, 180–240, and 240–300 m were respectively assigned with 20, 15, 10, 5, and 1, while for streams, buffer zones of 0–30, 30–60, 60–90, 90–120, and 120–150 m, respectively, with 10, 8, 6, 4, and 1. This implies that the closer to the river the higher the propensity of a landslide.

Finally, these buffers are converted to raster data with 30 m cell size using the “polygon to raster” tool as proposed by Wu et al. (2018) [30].

##### Geological and Geomorphic Data

(1) Geological strata and formations: Geological strata were extracted from the 1/50,000 Geological Map. Except for Ordovician, Silurian, Triassic, and Tertiary, the strata of other geological periods are mostly exposed. In terms of texture and composition, the lithology of different strata in the study area can be divided into 113 classes. To facilitate the geohazard analysis, these lithological classes were further aggregated into six main categories: (1) granitic rocks, (2) magmatic veins, (3) metamorphic rocks, (4) sandstone, (5) limestone, and (6) mudstone and shales as shown in Figure 4a. Based on lithology and in absence of faults and joints, granitic massif would possess the highest resistance to landslides while mudstone the lowest resistance. Hence, from (1) to (6), the propensity is likely to increase and these were respectively assigned values of 1, 2, 3, 5, 7 and 10.

According to field observations, landslide events occurred frequently on the boundaries between two formations, especially between the Quaternary sediments and other strata. Therefore, the lithostratigraphic boundary factor was also obtained by buffering and rasterization, then added to analyze the landslide susceptibility.

(2) Faults: This kind of geological structure has a prominent effect on the stability of rock mass [51,52]. In the study area there is a spectacular thrust nappe structure characterized by strong faulting activity. Such a structure is accompanied with a series of faults and folds, which tend to be the landslide-prone areas, e.g., the fragile belts related to fold hinges, fracture zones, and joints. As a matter of fact, the proximity to fault plays a role in such hazard events, i.e., the closer to the fault, the higher the propensity of a landslide. For this reason, the faults in the study area (Figure 4b) were divided into three groups in terms of scale, i.e., big faults if their length is >10–20 km, medium faults if they are 2–10 km, and small faults if they are <2 km. The big faults were buffered into five zones of 0–120 m, 120–240 m, 240–360 m, 360–480 m, and 480–600 m, and were respectively assigned values of 20, 15, 10, 5, and 1. The medium faults were also buffered into five zones of 0–60 m, 60–120 m, 120–180 m, 180–240 m, and 240–300 m with assigned values of 10, 8, 6, 4, and 1. The small faults were again buffered into five zones of 0–30 m, 30–60 m, 60–90 m, 90–120 m, and 120–150 m and respectively assigned values of 5, 4, 3, 2, and 1. These fault buffers were gridded into a raster layer of 30 m in resolution.
(3) Depth of the weathered crust, soil type, and texture: Weathering is the process of converting rocks into regolith and soils to constitute the weathered crust of our land surface. Landslides mostly take place in this crust in which soil texture seems to have a significant impact on [53,54] and the variability of soil types and depths of the crust play a part in the occurrence of such events [55]. Because different soil types and textures have different sand percentage, grain sizes and porosity affect the permeation of rain water. If liquidized by penetrated water, the crust bottom (soil/rock interface) may serve as a slip surface of a landslide as friction and resistance from the underlying rocks are reduced by this process. As soon as it has reached a certain threshold, a landslide occurs. Thus, the crust thickness, i.e., the depth of the slippery soil/rock interface, is a plausible indicator of landslide volume and scale.

The data of soil types were obtained from the Bureau of Jiangxi Coal Geology and the sand percentage (%), in which high sand percentage (low percentage of clay but high porosity) seems favorable for permeation of rain water and provoking landslide event, was considered as an indicator of soil contribution. Hence, soils with sand percentage >40%, 30–40%, 20–30%, 10–20%, 5–10%, and 0–5% were respectively assigned values of 10, 8, 6, 4, 2, and 1. Finally, the soil proneness map was converted into a raster of 30 m resolution.

The thickness data of the weathered crust were obtained from the field 1282 measurements. Assuming that all the ridges have a crust of 0.5 m in depth, these field-observed depths were interpolated using the kriging approach, then converted into a raster layer of 30 m resolution.

**Figure 5:**
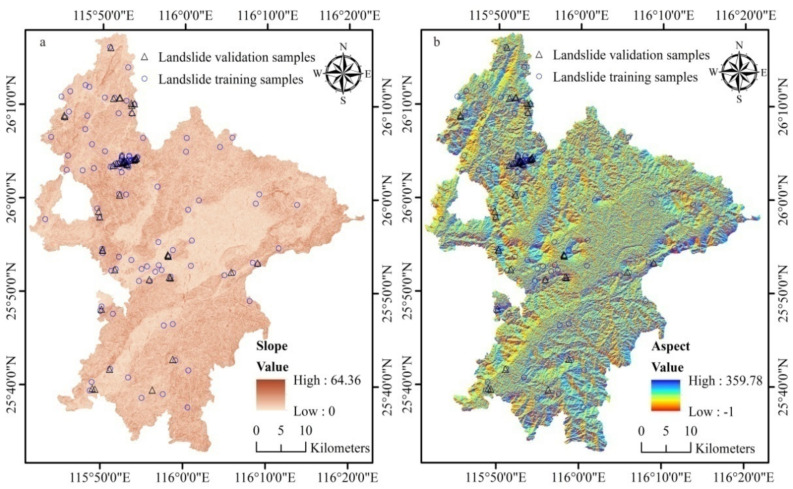
Geo-Environmental factors 3: (**a**) slope and (**b**) aspect.

(4) Geomorphic data: Slope (angle) is a key driver of landslides and a triggering angle threshold of 28°–38° was reported by Fan et al. (2016) [55]; at the same time, elevation, aspect, plane curvature, and profile curvature may also contribute to the occurrence of the hazards [14,21,56,57,58]. The ASTGTMV003 GDEM data, with a spatial resolution of 30 m, were obtained for Ruijin from NASA (www.earthdata.nasa.gov, 11 April 2020) and used to derive elevation, slope, and aspect (Figure 1 and Figure 5a,b).

##### Land Use/Cover, Transport System and Construction Sites

**Figure 6:**
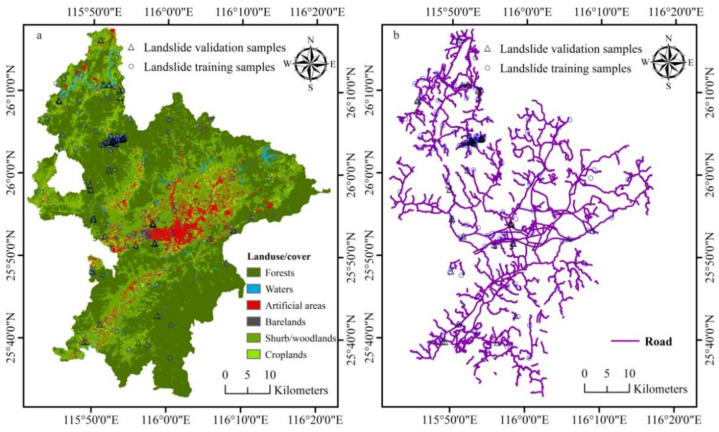
Geo-environmental factors 4: (**a**) landuse/cover and (**b**) road.

Using the classification approach proposed by Wu et al. (2016) [7], land cover mapping was achieved for Ruijin with an accuracy of 90.99%. The main land cover type is forests (54.25%), followed by shrub/woodlands (29.33%), croplands (6.65%), artificial areas (urban areas, villages, roads and other infrastructures, 5.36%), barelands (1.45%), and waters (1.41%) (Figure 6a). Forests cover hills and mountains; artificial areas and croplands are mainly distributed in lowlands (valleys) with low slope. For susceptibility modeling purpose, forest cover was considered of low proneness and assigned a value of 1. On the contrary, unvegetated hilly slopes and barelands were regarded as having a high propensity and assigned a value of 10, while zero-slope croplands, urban areas, and water-bodies were treated as no-risk (zero probability) areas. At the same time, NDVI can be used as an indicator of vegetation greenness and abundance, indirectly representing the development degree of the root system of forests and woodlands. For barelands, woodlands and forests, NDVI shall be a good indicator of propensity to landslide.

Road construction is one of the important human activities leading to slope failure [21,59]. Similarly, housing development located along two sides of roads or on the brink of hills by cutting slopes also constitutes an important factor that causes slope massif instability. The influence of roads on landslides is also reflected by distance to them [8,21,49]. The road system (Figure 6b) was assigned the same values as rivers and faults.

There were no landslide accidents recorded in the study area caused by earthquakes, so the latter was not considered as a triggering factor in this study.

#### 2.3.2. SNL Processing for Categorical Factors

SNL provides a digital label for each type of feature within the categorical variable. For example, instead of the above WA to each lithology of strata, each type of land cover, and each buffer of the linear factors, we gave an order number attributed respectively to the features of a given categorical factor or variable so that these factors were converted into numeric ones. After, they were rasterized.

#### 2.3.3. Frequency Ratio (FR)-Based Processing

The FR approach can be applied to calculate the relative impact degree of a given geo-environmental factor, either numeric or categorical, on a landslide event so that conversion of the categorical factors into numeric ones can be directly avoided [16,60,61]. In general, we have to divide continuous numeric factors into a number of subsets or intervals or consider each type of feature within a categorical factor as a “subset”. *FR* can be calculated by Equation (1):

(1) $$FR=\frac{N_{i}/N}{S_{i}/S}$$

where *N_i_* is the area of landslides occurring in the subset or interval of a given factor; *N* is the total area of landslides in the study area; *S_i_* is the subset or interval area of the given factor; and *S* is the total area of the study area. If *FR* is greater than 1, the possibility of landslide in this subset is high, otherwise, it is low [16].

#### 2.3.4. Integrated Datasets of Geo-Environmental Factors

The occurrence of a landslide is a result of the combined action of all the hazard-causative factors [14,19]. In this study, all these factors which may contribute to the occurrence of a landslide will be considered for susceptibility modeling. The raster layers, namely: geological strata; proximity to faults, lithostratigraphic boundaries, roads and rivers; thickness of the weathered crust, soil types and texture; elevation; slope; aspect; land use/cover; NDVI; multiyear annual mean rainfall; March–June rainfall; March–July rainfall; and May–July rainfall, of which the categorical factors were processed by SNL, were incorporated into a 19-band dataset (DS1) with Datum WGS 84 and Projection UTM 50 by the layer stacking function. Another 19-band dataset (DS2) was composed using the above geo-environmental factors in which the categorical ones were processed by WA approach, and the 19 FR-based raster layers constituted the 3rd dataset (DS3).

The raster layers in DS1, DS2, and DS3 were considered as hazard-causative factors or independent hazard predictors.

#### 2.3.5. Training and Validation Sets

As mentioned above, 155 landslides were obtained from the Geological Hazard Survey Campaign in Ruijin on a scale of 1/50,000 by the 264 Geological Team of Jiangxi Nuclear Industry in 2017. These landslides ranged from 20 m^2^ to 64,000 m^2^ in size and most of them are small in scale, i.e., less than 900 m^2^ in the study area. To obtain the optimal spatial presentation of the landslide samples for RF modeling, the landslides with areas less than 900 m^2^ were buffered with a radius of 30 m and then rasterized into pixels with a size of 30 m [30], and for those larger than 900 m^2^, a direct rasterization from the vectorized polygons was conducted. These cases were assigned a value of 1, indicating that the events of landslide have truly taken place, i.e., the probability is 1.

The selection of the non-landslide areas has an important influence on modeling landslide susceptibility, which was relatively easy to be ignored in previous studies. As mentioned above, the non-landslide stable areas, e.g., low-slope (<1–3°) croplands in valleys, plains, and urban areas were integrated into the field dataset as zero-risk areas, i.e., the occurrence probability is 0. Then, we randomly selected 70% of the landslide samples (109 cases) plus 70% of the stable zones (no risk) to constitute a training set (TS1) and used the remaining ones (46 cases, 30%) as a validation set (VS1).

Another approach to identify the stable area is to use *FR* calculation. The procedure is shown as follows:

Superpose the *FR* values of all geo-environmental factors to obtain the regional landslide susceptibility index (*LSI*) [16], which is calculated with Equation (2).

(2) LSI=∑FR

**Figure 7:**
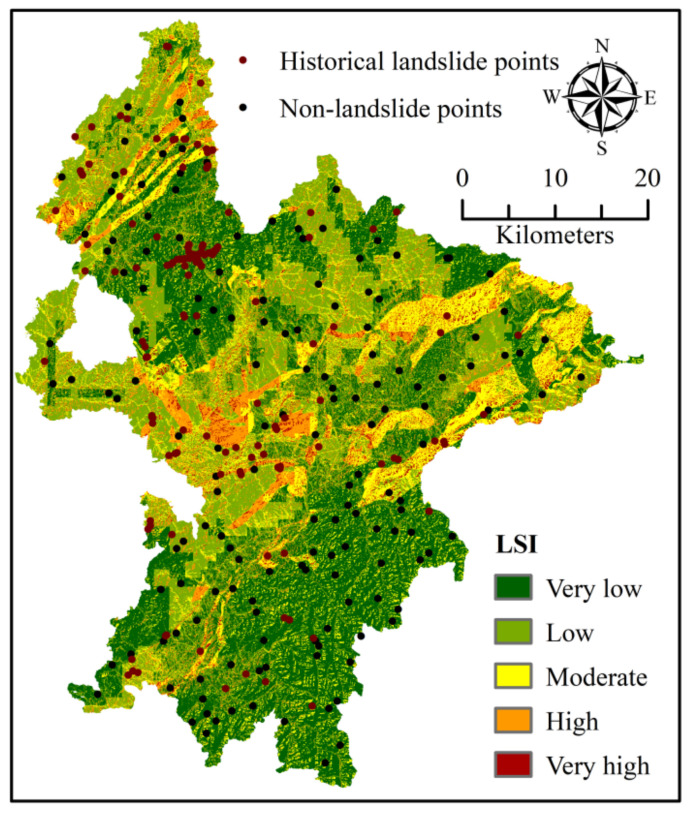
Landslide susceptibility index (LSI) of the study area and distribution of the non-landslide points.

Then, use this *LSI* (Figure 7) to identify the low-susceptibility zones, including both flat and non-flat areas, where the non-landslide (stable) points were randomly sampled. These new no-risk points were added into the observed landslides to generate another pair of training set (TS2) and validation set (VS2).

### 2.4. Landslide Susceptibility Modeling

Among the machine learning algorithms, Wu et al. (2016 and 2018) found that RF and SVMs performed equally well in classification, better than ANNs, but RF performed best in regression prediction [7,30]. Hence, the RF classification algorithm was selected for this modeling and SVMs for comparison. The overall procedure was already summarized in Figure 2 and the detail on modeling, validation, and accuracy assessment is given in the following subsections.

#### 2.4.1. RF Modeling of the Landslide Occurrence Probability

RF classification, based on growing decision trees, is an ensemble of tree classifiers that allow the classification of a given pixel by predicting its probability into the target class through majority voting. The key technique of this algorithm lies in that a bootstrap sampling of the TS is used to build each tree, and a stochastic selection of the input variables is searched to determine the best split for each node. Meanwhile, the RF algorithm uses out-of-bag (OOB) estimates to define the generalization error and the importance of each variable [37]. RF will not overfit if the number of decision trees (NT) increases to a certain level. Thence, NT should be large enough to reduce the OOB error of classification to a stable level in the training process. It should be noted that, instead of classification of land cover types, we employ this algorithm to classify the probability of landslide occurrence and non-landslide for each pixel.

#### 2.4.2. Application of the RF Algorithm

In this study, the RF classification was conducted within EnMap-Box which is a package particularly developed to process and analyze image data [62]. While conducting RF modeling, we regarded the three combined 19-band datasets, DS1, DS2, and DS3 as input predictive variables with TS1 and TS2 as dependent variables. Some key parameters of RF classification that require set up include the impurity function, the stop criteria (for node splitting), the number of randomly selected features (or number of variables) at each node and number of trees (NT) with the classification and regression algorithm [7,30].

The Gini coefficient was selected for the impurity function and the default value, i.e., minimum number of samples at a node of 1, was used for the stop criteria. The number of randomly selected features (or number of variables) at each node was the square root of all available features. The default value of NT was 100 within EnMap-Box. In this study, NT was set to 300 and 500 in order to achieve a better prediction.

After parameterization, or rather, modeling using TS1, the two derived RF models, namely, the SNL-based and WA-based RF models, were applied back to the integrated DS1 and DS2, respectively, for landslide prediction, i.e., the probability of landslide occurrence in each pixel, and VS1 was employed for validation of the models. While the FR-based RF modeling result using DS3 and TS2 was also applied back to DS3, and its accuracy of modeling was evaluated using the independent VS2.

#### 2.4.3. Importance of Variables

The importance of variables in the RF algorithm can be evaluated by the variable substitution method. In other words, it can be measured by calculating the difference of the OOB error before and after value substitution. The importance of factor *F_i_* can be expressed as follows:

(3) $$VIM\;(F_{i})=\frac{1}{NT}\sum_{t}errOOB_{t}^{i}-errOOB_{t},$$

where *NT* is the number of trees; *errOOB_t_* is an error for tree *t* of the forests when all the factors are included; *errOOB_t_^i^* refers to an error after removing the factor *F_i_*, and *VIM(F_i_)* is variable importance for *F_i_*. For the RF modeling and its result produced, a high value of *VIM(F_i_)* indicates the high importance of the factor *F_i_* or vice versa.

#### 2.4.4. Accuracy Reporting

Based on the confusion matrix, precision, recall, kappa coefficient (KC), and overall accuracy (OA) can be calculated to evaluate the accuracy and performance of the landslide susceptibility prediction model [63,64,65]. VS1 and VS2 were hence used to calculate these statistical indices. The evaluation results of TS1 and TS2 show the adaptability of the model to the training datasets while those of VS1 and VS2 reveal the predictivity and generalization ability of the models [66].

According to previous studies, the smaller the high-susceptibility area predicted by the model, the more historical landslide points are concentrated there, which indicates that the model has high reliability [21,34].

## 3. Results and Discussion

### 3.1. Landslide Susceptibility Maps

**Figure 8:**
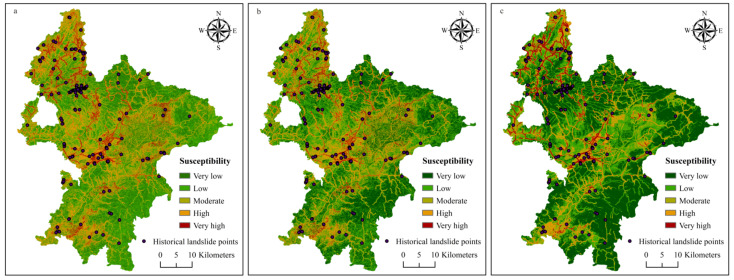
Landslide susceptibility zonation maps of Ruijin: (**a**) from the simple numeric labeling (SNL)-based RF modeling; (**b**) from the weight assignment (WA)-based RF modeling; and (**c**) from the frequency ratio (FR)-based RF modeling.

The landslide susceptibility zoning was achieved based on the modeled landslide occurrence probability when the RF modeling was implemented at NT = 300. Pixels in the study area were divided into five levels of susceptibility: very low (0–0.2), low (0.2–0.4), moderate (0.4–0.6), high (0.6–0.8), and very high (0.8–1.0). The landslide susceptibility maps of Ruijin were hence produced (Figure 8a–c).

The predicted results of the landslide-prone areas from the three schemes of data processing and sampling were quite similar and largely consistent with the field survey:

(1) Very high susceptibility zones were mainly linearly distributed along the roads and rivers due to the fact that a number of landslides were often caused by river undercutting and artificial road construction and housing development.
(2) In the central part of the study area, very high-susceptibility zones are concentrated in the Quaternary soil layer, or rather, in the weathered crust, especially along the boundaries of lithologic strata. The Quaternary unconsolidated soil layer with loose structure provided rich material for landslides. The boundaries of lithologic strata behaved as unstable structural interfaces, which appeared to be important factors for landslides.
(3) In the granitic massif, there were also obvious very high-susceptibility zones distributed along the roads. Weathering accelerated by humidity, high undulating landform and tectonically active settings of the study area change the intrinsic properties of the material and reduce the strength of the near-surface rocks.

As seen in Table 1, the zones of very high susceptibility generated by the SNL-based, WA-based, and FR-based RF models were 118.72 km^2^, 107.13 km^2^, and 135.32 km^2^, respectively, accounting for 4.86%, 4.39%, and 5.13% of the total study area. The high susceptibility zones of the SNL-based, WA-based, and FR-based RF models accounted for 437.27 km^2^ (17.92%), 363.78 km^2^ (14.91%), and 212.66 km^2^ (18.48%), respectively.

Additionally, 96.77% of the field samples, i.e., the real landslides, took place in 22.78% of the entire study area, which were categorized as high and very-high susceptibility zones in our zonation map generated by the SNL-based RF model. However, 93.55% and 96.13% of the field samples took place in 19.30% and 17.83% of the entire study area by the WA-based and the FR-based RF models.

It is noteworthy that 80% of the observed landslides fall in the scope of 0–120 m buffers of roads and new urban fringes, and 94.84% of the total landslides are related to human activities, e.g., development of roads and urbanization. This also reveals that the landslides constitute a significant risk to human society.

### 3.2. Number of Trees with RF Modeling

**Figure 9:**
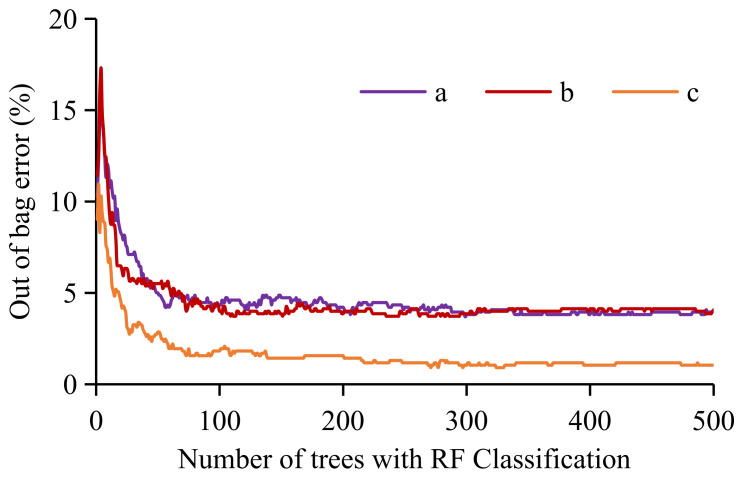
Out-of-bag (OOB) error plot versus number of trees (NT) with RF modeling: (a) simple numeric labeling (SNL)-based RF modeling using TS1, (b) weight assignment (WA)-based RF modeling using TS1, and (c) frequency ratio (FR)-based RF modeling using TS2.

The selection of NT has a great influence on the accuracy of RF modeling. The performance of classification or regression is poor and the error is large when NT is small. As it grows, the OOB error decreases continuously and eventually reaches a threshold [37]. However, the complexity of the RF models is directly proportional to NT. If there are too many decision trees, the operating efficiency will decrease as it becomes more time-consuming and the optimal result may not be obtained. The previous study by Wu et al. (2018) confirmed that in both low (e.g., 100) and high NT (e.g., 1000) cases, the algorithm did not perform well, but it did when NT was set to 300–500 [30]. It is clear that the OOB error tends to be stable after NT gets greater than 300 (Figure 9), or rather, the model accuracy becomes greater than 96%. Hence, 300 was finally used for NT when performing landslide susceptibility modeling.

### 3.3. FR and Importance of Geo-Environmental Factors

**Figure 10:**
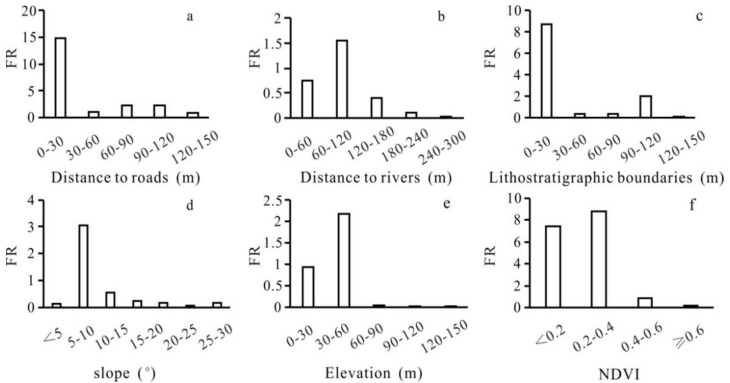
Frequency ratio (FR) of each geo-environmental factor: (**a**) distance to roads; (**b**) distance to rivers; (**c**) distance to lithostratigraphic boundaries; (**d**) slope; (**e**) elevation; and (**f**) NDVI.

The FR within each geo-environmental factor is presented in Figure 10. It is clear that the FR is negatively correlated with the distance to roads, rivers, and to the geological boundary and elevation, etc. This reveals the concrete role of each factor in the landslide event. Thus, the FR values of geo-environmental factors explain to a certain extent the importance of the independent variables demonstrated by the RF algorithm.

**Figure 11:**
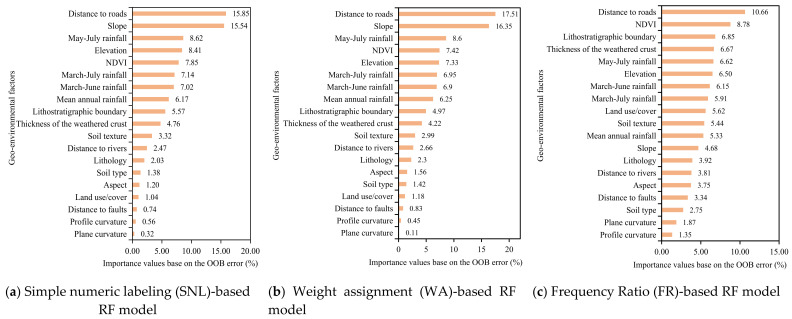
Importance (%) of the geo-environmental factors in landslide events from different random forest (RF) modeling.

In terms of the OBB error, the first five important factors of the SNL-based and WA-based RF modeling are as follows: (1) distance to roads, (2) slope, (3) May–July rainfall, (4) elevation or NDVI, and 5) NDVI or elevation (Figure 11a,b), while for the FR-based RF modeling, they are: (1) distance to roads, (2) NDVI, (3) Lithostratigraphic boundary, (4) thickness of the weathered crust, and (5) May–July rainfall (Figure 11c).

In the case of Ruijin, the order of importance seems plausible. A stable slope becomes unstable as a result of road construction, i.e., slope cutting or housing development. May–July rainfall shows most important among the different combinations of monthly rainfall, and can be regarded as a triggering factor as it liquidizes the slippery interface when it reaches a certain threshold, i.e., the rainfall amount leading to saturation of soil after penetration and starting to flow on the soil/rock interface. The more the rainfall in a short time, the higher the landslide susceptibility. Rainfall is thus widely employed as a weather indicator (WI) of landslides. NDVI, a late autumn mean of a five year period and an indicator of vegetation abundance, vigor, and root system development of forests and woodlands, can largely reflect the stability and instability of the weathered crust. It is hence reasonable that these factors were identified as the most important hazard-causative factors in Ruijin though all others may also play a certain role in geohazard events.

The importance of geo-environmental factors associated with landslides has also been discussed by other authors. Dou et al. (2019) showed that precipitation was the most significant factor, but according to those of Arabameri et al. (2017) and Cao et al. (2019), elevation was the most important factor [13,21,34]. It is understandable that in different geological environments, the mechanism of landslides may be different and so is the importance of geo-environmental factors.

### 3.4. Validation of the Modeling Results

Only after being validated, has the model potential to be applied elsewhere [29,63]. We used four statistical indicators to evaluate the performance of the landslide susceptibility model, including precision, recall, KC and OA as mentioned above. Against the VS1 and VS2, the statistical indicators of all three RF models were shown in Table 2. The FR-based RF model has the highest accuracy with KC and OA of 89.08% and 94.54%, respectively. The WA-based RF model obtained similar KC and OA results of 82.99% and 91.49%, respectively, followed by the SNL-based RF model with KC and OA of 79.26% and 89.61%, respectively. Those of the SVM models showed much lower results than RF models using the same predictive variables DS1, DS2, and DS3, and the same TS1 and TS2 (Table 2).

### 3.5. Case Verification

The 26 new landslides observed from 2019–2020 were used to verify the predictivity of the three RF models and we found that 15, 8, 2, 1, and 0 landslides are distributed respectively in the very high, high, moderate, low, and very low susceptibility zones for the SNL-based RF modeling map, while there were 15, 8, 3, 0, 0, and 15, 4, 5, 0, and 0 landslides in these zones for WA-based and FR-based RF modeling maps. From this point of view, WA-based RF modeling appears to have the best performance.

**Figure 12:**
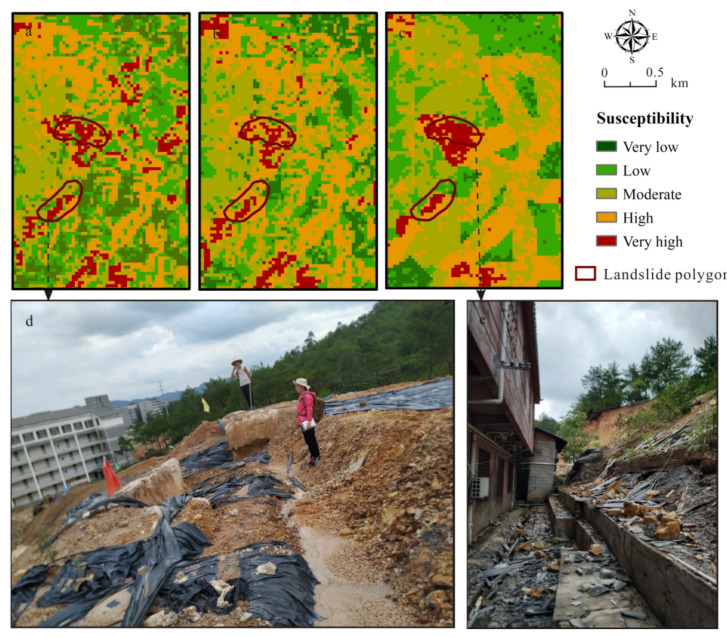
Prediction of the landslide susceptibility and case verification: (**a**) from simple numeric labeling (SNL)-based RF modeling; (**b**) from weight assignment (WA)-based RF modeling; and (**c**) from frequency ratio (FR)-based RF modeling. (**d**) landslide behind the No. 6 Middle School of Ruijin and (**e**) bulges on the side wall feet of the Longzhu Temple.

Surprisingly, the two large-scale creeping bedding slides (>20,000 m^2^) behind the newly-built campus of No. 6 Middle School and the Longzhu Temple in Ruijin that have been taking place for years, were well predicted as very high-susceptibility zones in the susceptibility map from the all three RF models (Figure 12a–c). During the field investigation in July 2019, the middle school was closed due to this disastrous effect (Figure 12d); just behind the Longzhu Temple there were significant ground bulges along the behind and side wall feet because of the extrusion provoked by the downward slide of the upper slope composed of the Carboniferous strata and its overlying Quaternary sediments (Figure 12e). Thus, both sites are in danger as landslides continue gradually and were reliably predicted by all these RF models.

## 4. Conclusions

The prediction and prevention of landslide disasters is essential to secure our society. This research illustrates that the combination of remote sensing, geological, geomorphic, climatic, and human dimensional data is relevant for such geohazard susceptibility zoning and mapping, and the RF algorithm has performed better than SVM in this case.

This paper presents three different processing schemes for the multi-source geo-environmental factors. We found that FR-based RF algorithm shows slightly better prediction accuracy, but WA-based RF model is of slightly better predictivity and able to derive more reliable results. We believe that our research will be helpful for local government to act on the prevention and early warning of geohazards to ensure people’s safety and property, and to provide theoretical advice for the infrastructure construction and urban planning. In the next phase of our work, a dynamic monitoring and early warning system will be designed and implemented in the very high and high susceptibility zones predicted by the above models. To achieve this, geodetic data obtained from the Global Navigation Satellite Systems (GNSS) for displacement and deformation monitoring will be essential and ground monitoring stations/equipment will be installed in the above critical areas with help of the Internet of Things and micro-electro-mechanical systems (MEMS).

Our study also reveals the critical role of human activity, in particular, road construction and housing development in landslide events. Most of the observed landslides in Ruijin were actually “man-made”. In future, when we design road system development and effectuate new urban planning, we should assess the negative impacts.

Another innovation lies in identifying three approaches for conversion of the categorical geo-environmental factors, such as geological strata, faults, soil, roads, and rivers into numeric ones, for example, WA-based, SNL-based, and FR-based so that quantitative susceptibility modeling and prediction using the RF algorithm can be smoothly achieved. We would like to highlight that both SNL-based and FR-based processing is able to avoid subjective weight assignment, but WA-based RF modeling may lead to the most reliable prediction. Therefore, this study can serve as a prototype for similar research elsewhere.

## Figures and Tables

**Table 1:** Distribution of landslides within different susceptibility levels.

RF Model	SNL- Based	WA- Based	FR- Based	SNL- Based	WA- Based	FR- Based	SNL- Based	WA- Based	FR- Based	SNL- Based	WA- Based	FR- Based
Susceptibility Level	Area (km^2^)			Percentage (%)			Number of Historical Landslides			Percentage (%)		
Very High	118.72	107.13	135.32	4.86	4.39	5.13	132	137	135	85.16	88.39	87.10
High	437.27	363.78	212.66	17.92	14.91	12.70	18	14	14	11.61	9.03	9.03
Medium	665.71	545.69	364.47	27.28	22.56	18.79	3	1	5	1.94	0.65	3.23
Low	726.33	745.11	679.71	29.76	30.53	25.27	1	2	1	0.65	1.29	0.65
Very Low	492.35	678.68	1048.24	20.18	27.81	38.12	1	1	0	0.65	0.65	0.00

**Table 2:** Performance of the RF and SVM algorithms vs. validation sets (VS1 and VS2).

Item	SNL-Based RF Model (VS1)	WA-Based RF Model (VS1)	FR-Based RF Model (VS2)	SNL-Based SVM Model (VS1)	WA-Based SVM Model (VS1)	FR-Based SVM Model (VS2)
Precision (%)	94.67	95.00	94.00	83.33	84.67	92.67
Recall (%)	85.54	88.67	95.27	82.78	83.55	77.65
KC (%)	79.26	82.99	89.08	63.37	65.50	66.00
OA (%)	89.61	91.49	94.54	81.79	82.86	83.00

## Data Availability

The data that support the findings of this study are available from the corresponding author, Weicheng Wu, upon reasonable request.
